# Exploring community tuberculosis program in the pastoralist setting of Ethiopia: a qualitative study of community health workers’ perspectives in Borena Zone, Oromia Region

**DOI:** 10.1186/s12913-021-06683-y

**Published:** 2021-07-01

**Authors:** Abebe Megerso, Negusie Deyessa, Godana Jarso, Robel Tezera, Alemayehu Worku

**Affiliations:** 1 Department of Public Health, Adama Hospital Medical College, Adama, Ethiopia; 2 Department of Preventive Medicine, School of Public Health, Addis Ababa University, Addis Ababa, Ethiopia; 3 Department of Medicine, Adama Hospital Medical College, Adama, Ethiopia; 4 Department of Radiology, School of Medicine, Addis Ababa University, Addis Ababa, Ethiopia

**Keywords:** Case identification, Community TB, Community Health workers, Pastoralist

## Abstract

**Background:**

Pastoralist community accounts for a significant portion of the population in Ethiopia. This community is different from majority of the country’s population. Access to TB prevention and control services is uneven in the country. The community TB program is designed to improve the access. Exploring the program performance from the perspectives of its implemters in a pastoral setting remains important.

**Method:**

We conducted a qualitative study using an interpretive description method in the pastoralist community setting of Ethiopia. Study participants were recruited from geographically dispersed areas. We collected data through in-depth interview using semi-structured interview guides and audio recordings during February 01–30, 2020. The guides were developed in consultation with TB program experts and clinicians treating TB patients in the study area. Notes were taken at the interviews to enrich transcription of the data. Principal investigator conducted the interview. The subsequent interviews were informed by emerging ideas from forgoing interview transcriptions and continued until data saturation was achieved.

**Results:**

One hundred and fifty six codes, nine categories and three themes emanated. The first theme was inadequate community TB performance and some of its codes include inadequate presumptive TB case identification and compromised directly observed treatment short course service delivery. The second theme was factors contributing to the program performance. Community factors, lack of physical access to health facilities and indirect non-medical cost were some categories under this theme. The final theme was suggested solutions; and its categories include a need for active community involvement and modification of service delivery approaches.

**Conclusions:**

Community TB performance was inadequate in the pastoralist community. Multifaceted factors contributed to the inadequate program performance. Socioeconomic and access related factors were major contributers. Aligning the program to the context of the pastoralist community setting is required to improve the performance.

## Background

Tuberculosis (TB) have been declared to be a global public health emergency by the world health organization (WHO) nearly three decades ago in 1993. Human immunodeficiency virus (HIV) and the emergence of multidrug resistant TB (MDR TB) fuelled the pandemic and complicated its prevention and control efforts [1]. ‘END TB’ strategy was developed in 2014 with targets to reduce TB incidence by 50 % (to less than 55 TB cases per 100,000 population) and reduce TB deaths by 75 % compared with 2015, and no affected families facing catastrophic costs due to tuberculosis by 2025 [2]. However, TB was one of the top 10 causes of death worldwide and the leading cause of death from a single infectious agent (ranking above HIV/AIDS) in 2019 [3]. Globally, an estimated 10.0 million people fell ill with TB in 2019 and there were an estimated 1.2 million TB deaths among HIV-negative people, and an additional 208 000 deaths among HIV-positive people during the year. Despite increases in TB notifications, there was a large gap between the number of new cases reported and the estimated incident cases in 2019 as it was the case during previous years. Access to universal health care, including TB case identification and treatment, is still falling short of the intended health coverage [3]. Africa contributed the second largest percent of TB cases (24 %), next to the South-East Asia WHO regions, to the world’s TB cases in 2018. Ethiopia is among the 30 global high TB burden countries and one of the ten triple burdened (TB, TB/HIV and MDR TB) countries in the world [4]. The country adopted WHO strategies and guidelines to combat and reverse the TB transmission [1, 5]. TB case, the notification is neither adequate for WHO estimated incident cases nor consistent across local diversities in the country due to variation in access to TB prevention and control services [6]. Pastoral community is one of the most marginalized settings in the country, particularly in regions where the agrarian community is predominant [7]. But, pastoral communities occupy 43 % of the land mass of Africa and Ethiopia is one of 36 countries with large area of the pastoralist livelihood community [8]. Pastoralism is a culture, livelihood system, extensive use of rangelands. It is the key production system practiced in the arid and semi-arid dryland areas. Recent estimate sindicate that about 120 million pastoralists and agro-pastoralists life worldwide, of which 41.7 % reside only in sub-Saharan Africa [9]. Pastoralists live in areas often described as marginal, remote, conflict prone, food insecure and associated with high levels of vulnerability.

Pastoral communities of Ethiopia occupy 61 % of the total land mass and 97 % of Ethiopian pastoralists found in lowland areas of Afar, Somali, Oromiya, and Southern Nations Nationalities and peoples’ region. In spite pastoralareas have a significant role in the national economy, yet very little consideration was given to pastoral development and policy makers often neglect them, focusing on the interests of agriculture and urban people [7]. The health system of Ethiopia was highly centralized and access to basic health services was very poor before 2003. Among other efforts to improve the access, the country has been implementing a health extension program (HEP) since the year 2003 where community health workers (CHWs), called health extension workers provide packages of primary health care activities at community level [10, 11]. The CHWs are those who have attended basic education and trained on primary health care packages at least for one year after completing high school education. Implementing the program enabled Ethiopia to achieve significant improvements in maternal and child health, communicable diseases, hygiene and sanitation, knowledge and health care seeking [11, 12]. The community TB program is one of the service packs implemented by community health extension workers (CHWs). In spite of these successes, the program is currently facing challenges. These challenges are related to productivity and efficiency of the CHWs, working and living conditions of the CHWs; capacity of health posts and, other social determinants of health [13]. As part of the communicable diseases prevention and control package, TB prevention and control activities have been decentralized to the community level, through community TB program, where CHWs provide short course directly observed treatment (DOTs) in health posts, the most peripheral health facility, located in the smallest administrative unit called Kabul. Besides the DOTs service provided in this health facility, CHWs are responsible for presumptive TB case identification and referral, provision of health education and adherence support among other activities by conducting regular home visits. The CHWs, the health extension workers, are female (only a few of them working in hard to reach areas are male) and most of them have families including young children. In the pastoral community setting, where CHWs are engaged in the prevention and control of TB, including identifying presumptive TB cases and referring them to health facilities for diagnosis along with other primary health care services, exploring the community TB program to understand the performance in terms of case identification and DOTs services provision, assessing factors underlying the performance and pointing out solutions from the perspectives of the implements (CHWs) is important.

## Methods

### Setting

This study was conducted in a pastoral community of the Borena zone in Ethiopia. Pastoralist community covers 12–15 % of the total population of the country [9, 14, 15]. In Oromia Region, pastoralist community constitutes 43 % of the land mass and 16 % of the regional population [16]. Borena zone is a pastoral community area of the region and shares boundary with Kenya in the south and Somali regional state in the east neighbourhood by the similarly pastoral community. The community is highly mobile for the search of pasture and water for livestock, lives in scattered settlements and its access to health facilities is limited. Due to the settlement pattern, even the lowest health facility, the health post, is far from most community members. In this zone, 531 CHWs, of which more than 95 % are female, provide primary health care services at health posts to over 700,000 population and work on disease prevention activities including TB prevention and control that they perform by conducting regular home visits.

### Study Design

In this study, we used an interpretive description method which is one form of the generic qualitative research approaches [17, 18]. This approach is not guided by any of the explicit set of philosophical assumptions that underlie designs such as phenomenology, grounded theory, and ethnography. Generic qualitative studies exhibit some or all of the characteristics of qualitative endeavour, but rather than focusing the study through the lens of a known methodology, they seek to either combine several methodologies, or claim no particular methodological viewpoint at all [17]. This approach is preferred when the research question can not be directly aligned with any of these established qualitative study designs [19, 20]. In this study, we used a generic approach which claims no particular methodological viewpoint of the other established designs.

### Recruitment Strategy

Study participants were CHWs, called Health extension workers in Ethiopia. We recruited health posts haphazardly from geographically dispersed areas, in terms of distance and direction from the centre, of Borena zone. In the selected area, where there were more than one CHWs available, one with longer work experience was selected purposively for the interview. This approach is believed to facilitated unbiased selection of the study participants as the Borena zone is a homogeneously pastoral area with almost similar livelihood and health care access. In cases where two or more community health worker work in the same health post, one of them was interviewed. Among them, one with long work experience of the area was given priority to be interviewed for better information.

### Data Collection

Data were collected through in-depth interview using semi-structured interview guides (Table 1) and audio recording during February 01–30, 2020. The interview guide was developed for this study in consultation with TB program experts and clinicians treating TB patients in the study area. Each in-depth interview lasted for 20 to 40 min. Notes were taken at the interview to enrich the transcription and translation of the data. The interview was conducted by the principal investigator. It was conducted in local languages spoken by the participants, that is Oromo language. The interviewer introduced himself clarifying that he has no any direct responsibility in the health system to minimize social desirability bias of the study participants [21]. He has no any relationship or conflict of with the pastoral community perse. The subsequent data collection was informed by emerging ideas from forgoing interview transcriptions as both activities were happening simultaneously. To ensure the transcript content concurrence with that of the interviewee, members checking was done with five interviewees. We shared respective transcripts with the interviewee, received comments and ensured the concurrence. Study participants recruitment and interview continued until data saturation is achieved [22–24].

**Table 1:** Interview Guides (Detailed probing questions not included)

1	In general, how do you explain the implementation of the community Tuberculosis program in your catchment area? Please, give us details of the strategies you use to achieve the program objectives.
2	How do you see your performance in achieving the program objectives and plans? Please, explain to me major plans you have and your achievements.
3	We know that early identification of presumptive TB cases, linking them to health facilities for diagnosis and treatment and providing DOTs services at health posts are few of the TB prevention and control program activities. Can you explain achievements in these activities?
4	Now, let’s talk about factors that contributed to your performance in implementing the community TB Program. Tell me what factors affected you to or not to perform well in your activities? I wonder if you can elaborate your ideas by your own experiences!
5	As a community health worker engaged in the program, your suggestions can serve as first hand information for the program improvement. What do you think should be done to improve the community TB program? Please, explain your suggestions indicating who should do what as broadly as possible.

### Data Analysis

Data analysis was done concurrently with the data collection using a Microsoft office word processor [25]. Interviews conducted on a day were transcribed from the audios and notes taken in the field before the next data collection. Information from the analysed data was used to enrich the subsequent interviews. Independent coding was done by two authors to keep validity and reliability of the theme development [26]. Consistency between the audio and the transcriptions was checked by two of the authors (author one and three), who speak the language used in the interview, independently. Furthermore, the audio records and transcripts were cross-checked by independent experts before the final analysis. Finally, all authors checked and agreed on the development of code categories and themes.Our study participants (CHWs) have embedded knowledge of the pastoralist communities and also understand the health system of the country, Their perspectives on community TB program performance are important to leverage in designing appropriate solutions for these unique marginalised communities. With this premise, we analysed the interview transcripts and audio records without requiring other data sources for triangulation.We followed an inductive content analysis approach [27]; that is identified codes, sorted into categories and developed themes which finally synthesized. We used a combination of ontological (capture participant realities) and epistemological (understand the phenomenon) approaches to explore community TB programs in the study setting. An interpretive description method was used to analyse the manifest content, Furthermore, a hermeneutic interpretation method was used to analyse latent contents [18, 19, 28]. The findings were reported using textual descriptions and quotes to illustrate ideas and illuminate experiences [29]. We also produced a concept map that shows the interrelation between themes emanating from the analysis. 

## Results

Twenty two community health workers, also called health extension worker, who had three or more years of work experience at the time of interview were included in the study. The age range of the interviewee was between 20 and 30 years. Eight of them were trained to level four while the other were to level three (Table 2). From the analysis of interview transcriptions, 156 codes and nine categories emanated which were finally organized into three themes. The first theme was community TB performance. Under this theme, inadequate presumptive TB case identification and DOTs service compromise were the two categories emerged. The second theme emanated from the data was factors contributing to the first theme. Under this second theme, five categories such as community, physical access, CHWs, directed non-medical cost and referral health facility related factors emerged. The final theme was related to solutions. The two categories under this theme include active community involvement through the use of community own structure and need for the modification of service delivery approach (Table 3).

**Table 2:** Socio-demographic characteristics of study participants, Borena zone of Oromia Region, Ethiopia in 2020

Participant code	Age Category (yrs)	Sex	Marital Status	Educational Status	District
CHW-1	20–25	Female	Married	Level III	Elwoye
CHW − 2	20–25	Female	Married	Level IV	Dire
CHW − 3	20–25	Female	single	Level III	Elwoye
CHW − 4	26–30	Male	Married	Level III	Elwoye
CHW − 5	26–30	Female	Married	Level IV	Dire
CHW − 6	20–25	Female	Married	Level III	Yabelo
CHW − 7	20–25	Female	Married	Level III	Yabelo
CHW − 8	20–25	Female	Married	Level III	Yabelo
CHW − 9	20–25	Male	Married	Level IV	Dass
CHW − 10	20–25	Female	Married	Level IV	Dass
CHW − 11	20–25	Female	Married	Level III	Dass
CHW − 12	26–30	Female	Married	Level III	Moyale
CHW − 13	20–25	Female	Married	Level III	Moyale
CHW − 14	20–25	Female	Married	Level IV	Moyale
CHW − 15	20–25	Female	Married	Level IV	Melka Soda
CHW − 16	26–30	Female	Married	Level IV	Melka Soda
CHW − 17	20–25	Female	Married	Level III	Melka Soda
CHW − 18	20–25	Female	Married	Level III	Dubluk
CHW − 19	20–25	Female	Married	Level IV	Dubluk
CHW − 20	20–25	Female	Married	Level III	Dubluk
CHW − 21	26–30	Female	Married	Level III	Arero
CHW − 22	20–25	Female	Married	Level III	Arero

**Table 3:** Emanated Themes, Categories, Codes and respective frequencies, Borena zone of Oromia Region, Ethiopia in 2020

Themes	Categories	Codes (Frequency)
**Low Performance**	**Inadequate Case Identification**	Low number of cases detected (13)
	**Compromised DOTs**	Providing drugs for days (6)
		Awareness(22)
		Perception(6)
	**Community Factors**	Discrimination (4)
		Acceptance to CHWs (8)
		Boarder conflicts (4)
		Distance to referrral health facilities (16)
**Contributing Factors**		Distance from households to HP (14)
	**Physical Access Factor**	Scaterred settlement (12)
		Pastoral livelihood (6)
		Lack of transport facility (5)
		Difficult wheather condition (9)
		Commitment to work (3)
	**CHWs related Factors**	Distant living area (4)
		Work overload (2)
	**Direct non medical Cost**	Transport cost (6)
	**Referral HF related Factors**	Long Lab test turnaround time (4)
	**Community Involvement**	Using Community own structure (8)
**Suggested Solutions**		
		Outreach services with HCs (2)
	**Modified Delivery Approach**	EstabilishTB sputum slide fixing at HP (2)

Each of the themes contained a wealth of information that can contribute to better achievement in the TB prevention and control program in the pastoral community in general and current study setting in particular. The information is organized by theme and categories supported by selected quotes taken from the interview.

### Performance status

Presumptive TB case identification and provision of DOTs services are two of the key community TB program activities performed by the community health workers. These two activities were used as the program performance indicators in this study.

#### Inadequate Presumptive TB case Identification

Inadequate presumptive TB case identification was one of the categories emerged in the study under the main theme of low performance of a community TB program. Most study participants emphasized that they could not identify adequate number of presumptive TB cases and link to health facilities for diagnosis and treatment. One of the interviewees stated their performance as follows:

*… Our performance in identifying presumptive TB cases is low compared to expected number of cases. Although there are people with some symptoms of TB, such as cough of two weeks or longer, they do not accept our referral to health facility for lab tests. They say, ‘we do not need to go to a health facility. The cough will not be that bad to require us to go to a health facility’… (****CHW****5)*.

#### Compromise in the provision of DOTs services at health post (HP)

Community health workers are responsible to provide DOTs services at their respective HPs. Patients residing in the catchment area of the HP who are started on anti TB treatment in higher heath facilities such as health centre and hospitals are referred to their respective HPs for the treatment refilling and adherence monitoring. As per the national guidelines of the country, the drugs are given in the morning under the supervision of CHWs. In this study, many study participants witnessed the compromise in the DOTs service provision due to multiple reasons to be presented in the subsequent parts of the study. Examples of quotes that elaborate the theme and categories under it, taken from the statements of the study participants, are given below (Table 4).

**Table 4:** Key Theme: Low community TB program performance, Borena zone of Oromia Region, Ethiopia in 2020

***Sub Themes Examples of Quotes from Participants***	
Inadequate presumptive TB case Identification	*… we work to to identify presumptive TB cases such as those having cough of two weeks or more and refer them to health facilities for diagnosis. We have expected number of cases to be referred in a year. Regardless of our efforts to identify the cases, our achievement is lower than expected as some cases we identified decline our referals … (****CHWs − 6****)*
Compromised DOTs service	. *Providing DOTs service every morning at our health post (HP) is one of our tasks. To perform this test, I have to be available in the HP for quite a long time to wait patients coming from the community. This is difficult as we also need to conduct home visits and do other activities in the community. For this reason, sometimes, we give drugs to the patient for a week, although we know that adherence to the treatment might be compromised… (****CHW-5****)*

NB. (CHW-6) and (CHW-5) are study participants’ identifiers

### Factors contributing to the inadequacy of performance in Community TB program

From the total 156 significant information or codes identified, 125 were categorized under the five categories of this theme. The five categories, emerged under this theme and each of which contain a number of codes were community, physical access, CHWs, direct non medical cost and referral health facilities related factors. Study participants mentioned a lot of information corresponding to each category (Table 5).

#### Community Related Factors

Many significant information emanated under this category. These include low community awareness of TB, perceived severity of the disease, acceptance to health education given by CHWs, discrimination to TB patients, occasional boarder conflicts which results in instability of the settlement of the community. These all factors synergistically contribute to poor performance of the community TB program.

#### Physical access to Health facilities

Although the purpose of designing community health extension program, including the community TB is to ensure universal access to primary health and essential health care services, the intended decentralization is not yet achieved in the pastoral community setting. In this study, a number of codes emanated from the data. The codes include distance to referral health facilities from the community, distance from households to HP, scattered settlement of the community villages, pastoral livelihood (mobility in search of pasture and water), lack of transport facility, difficult weather condition and hard to reach catchment population.

#### Community Health workers related Factors

A couple of codes emerged related to the CHWs from the analysis. One of them was the lack of commitment to the work by the CHWs. That is, some of the CHWs got disappointed with multiple challenges they have been facing and lack improvement in the situation, they tend to have minimal courage to work to achieve the intended goals of the program. Most CHWs do not have at least locally appropriate living room in the work area and daily travel from distant living area. Most of them are female and have their own children to care. Work overload is another repeatedly mentioned code under this category. They have to conduct community home visits and provide selected services at the HP including DOTs services which should be given on a daily basis. In such a pastoralist community, mothers have much responsibility to take care of their children besides the health service provision to community in a setting. 

#### Direct non-Medical cost elated Factors

The transport cost is one of the most frequently mentioned significant information under this category. In the community where health care seeking practice is low and lack of transportation facility. Unaffordable transport cost if they find one, is major problem contributing to poor performance of the community TB in the current study setting.

#### Referral Health Facilities related Factors

Laboratory facility, long Lab test turnaround time and lack of appropriate health care provider in the referral health facilities are significant information mentioned in this category. These conditions are believed to discourage presumptive TB cases from accepting referrals from the CHWs.

**Table 5:** Key Theme: Factors Contributing to the low community TB performance, Borena zone of Oromia Region, Ethiopia in 2020

***Sub Themes***		***Examples of Quotes from Participants***	
Community related Factors		. *Many community members consider TB related signs and symptoms as mild and self limiting complaints and do not accept our referral to health facilities. Some members miss understand our messages about TB prevention methods and tend to discriminate people who have cough or on anti TB treatment… (****CHW-12****)*	
Physical access to Health facilities		… *We do not have the facilities to test for TB in this health post. We do not have the skill and material to take and send samples to health facilities either. We just inform people with sign and symptom of TB and give a referral slip to go to other health facilities for the test. The health facilities are bout 40 Kms from here and also there is no transportation facility. Not only diagnostic health facility, but our health post is far from villages. Therefore, access to health facilities is inadequate in our pastoral community and there is a need to revise TB prevention and control approach in our setting… (****CHW-16****)*	
Community Health workers related Factors		*… Most of us (CHWs) are female. Pastoral community settlement is highly scattered and mobile from place to place in search of pasture. Some of us have their own baby to care for and living place nearby health post is not good or non existent either. Travelling long distance on foot in this hot and hard to reach areas is difficult and we are unable to perform as required unless some solution is given from concerned bodies… (****CHW-1****)*	
Direct none medical cost related Factors		*… Due to the distance of the referral health facilities and lack of transport facilities presumptive TB cases have to pay money which they cannot afford for the transportation to health facilities for laboratory test. As we refer them based on signs and symptoms, sometimes laboratory tests turn out to be negative. In such cases, referred people complain that we caused them to incur unnecessary cost and this affects acceptance health education messages and referral…. (****CHW- 4****)*	
Referral Health Facilities related Factors		… *Referral health facility to which we refer has its own problem. Sometimes, the referral health facilities are not provided the requires services for various reasons, some which are lack of responsible health worker and lack laboratory supplies. Even when available, the laboratory result turn around time is not short enough and people coming from far villages can not wait and may need a second day return. This incurs additional cost to the patients and discourage them affecting health care seeking… (****CHW – 10****)*	

NB. (CHW-12), (CHW-16), (CHW-1,) (CHW-4) and (CHW-10) are studied participants’ identifiers

### Suggested Solutions

Community health workers are the actual implementors of the community TB program. Noticing solutions they suggest is important for the program. Under the theme of suggested solutions, two categories of significant information emerged. Each of the categories is presented as follows (Table 6).

**Table 6:** Key Theme: Suggested Solutions (from CHWs perspectives), Borena zone of Oromia Region, Ethiopia in 2020

***Sub Themes***			***Examples of Quotes from Participants***	
Active Community involvement ( Use of community structure)			*… In this pastoral community, each village has its own cultural leaders and all members of the village accept issues presented to them by the leader. The members are more obedient to such leaders than the kebele nominated people to the village, involving such leaders in the TB prevention and control program including TB treatment support will help the program… (****CHW − 8****)*	
*Modified service delivery approach*			*… We are working in this community for quite a long time. The community is not accepting our health education regardless of our efforts. I think it will be better if we work with health centre workers. It is also good to have a slide fixing facility in the HP so that patients will not incur cost of transportation. I hope such access facilitation will improve the program performance… (CHW − 14)*	

NB. (*CHW- 0*) and (CHW − 14) are studied participants’ codes given in Table 1

#### Community Involvement

Pastoral community has its own structural organization. For example, in the current study setting, the community have village specific community leaders and methods of communication. Local leaders are highly acceptable by the community members. Therefore, using Community own social structure helps to realize active involvement of the community members and hence important to communicate necessary health education messages. This community involvement can be extended to the level of starting home based TB treatment delivery with the close supervision of trained treatment supporters selected by the community.

#### Modified Delivery Approach

The other category, emerged under this theme, was the need for modification of community TB services approach. In this category, significant information repeatedly mentioned by the study participants includes further decentralization of the activities such as outreach services to be conducted on a regular basis in coordination with health centre staff and establishing TB sputum slide fixing at the HP. If appropriate capacity building training is given, CHWs can take samples and send the specimen for laboratory testing so that direct non-medical cost to patients could be averted and in turn, access to the service will be improved.

### Summary and Synthesis of the Themes, Categories and Codes

**Fig. 1:**
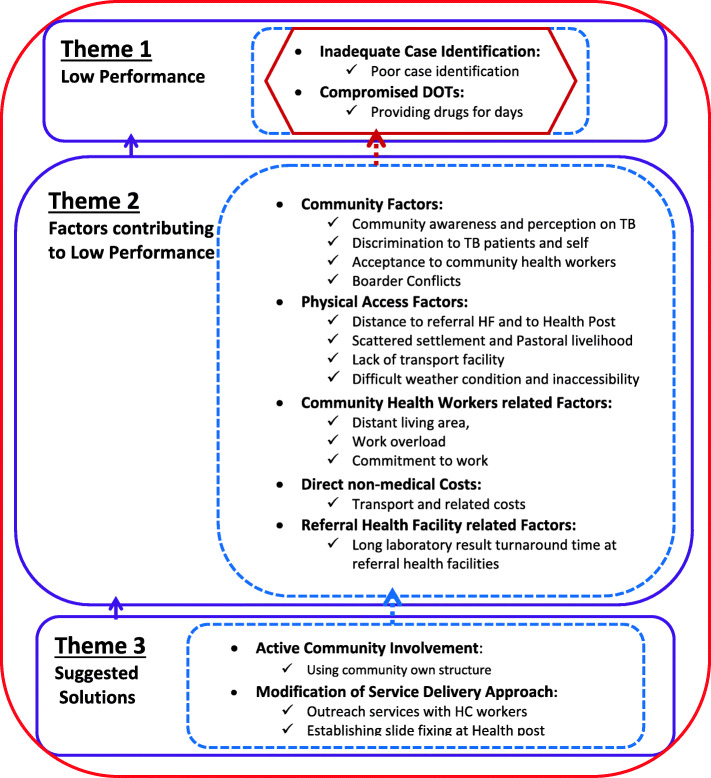
Summary and Synthesis of the codes, categories and themes, Borena zone of Oromia Region, Ethiopia in 2020

The above five categories of codes are organized and finally three themes emerged from the analysis. The three themes, interplays between sub-themes and their implications were illustrated using a diagram (Fig. 1).

## Discussion

Decentralizing health care services, through the implementation of health extension program, including community TB, was designed to improve access to the primary health care services. In this study, we explored the performance of the community TB, which is one of the packages decentralized to the CHWs in the pastoral community setting. The first theme emerged in this study was the low performance where CHWs’ contribution in presumptive TB case identification and DOTs provision is not achieving the goals. One indicator of the poor performance was inadequate presumptive TB case identification and referral which consistent with other study reports [30, 31]. Inappropriate community TB approach where DOTs is highly compromised may lead to poor adherence to anti TB treatment [32, 33]. This compromization of the DOTs service can in turn lead to the development of drug resistance by Mycobacterium TB bacteria which is emerging challenge to TB prevention and control. In general, performance of community TB in the pastoral community is under expectation consistent results of other studies [34, 35]. In the main theme of factors contributing to inadequate community TB performance, one of the categories emerged was community related factors such as awareness on TB, perception, acceptance to CHWs and discrimination of TB patients. These findings, particularly the influence of community awareness on TB, are consistent with reports from other studies in Africa [14, 32, 32, 33, 36]. The other identified factor contributing to the inadequate community TB program performance, problem in physical access to TB care facilities, is in agreement with reports from other studies [14, 35]. This problem was mentioned by TB patients themselves in our previous study conducted in the same setting [37]. Factors related to CHWs such as work overload, lack of conducive work environment, including home to live in close to the HP, and related demotivation by the CHW, which are important findings, were similarly reported in earlier studies [30]. The other categories emerged under this theme were direct non-medical cost and referral health facility related bottlenecks. These findings were similarly identified in earlier studies, although the extent could be worse in the pastoralist community setting due to other related conditions such as poor access to health care facilities [35, 38, 39]. The third theme, identified in this study, was about how to improve or align the community TB program with the pastoralist community setting. Based on the inadequate performance of the program and identified contributing factors, the CHWs who are actual practitioners of the program in the study setting, have suggested the need for customization of the approach to community setting which is consistent with other studies recommendations [31, 40]. One of the categories emerged under this theme was the need for use of community own traditional organization. Pastoralist community has well organized and functional social organization. This social organization in which the community has its own leaders in each village and settlement can help the community TB program comprehensively and this recommendation is consistent with findings of other studies conducted in similar settings [32, 34, 41, 42]. This can insure active community involvement and ownership [43]. The other solution proposed by the CHWs was a need for modification of the service delivery approach. These include the need for active engagement of health centre staff through planned outreach campaigns for TB prevention and control activities and enhanced support to the CHWs. Furthermore, there is a need for CHWs capacity building trainings to enable them to collect and fix sputum samples so that referral related challenges of the cases will be elevated. This is consistent with other studies findings [14, 32, 41, 43]. It is also suggested by the CHWs that there is a need to further TB treatment decentralization to facility level where trained treatment supporters can work in collaboration with the CHWs. This approach was also reported to be helpful in settings similar to our study area [34, 42, 43]. In summary, as TB its self and its interventions are affected by various cotext specific socioeconomic determinants, our study brought up important findings which can inform relevant stakeholders. Findings from the study indicated that community TB program performance is very low in the current setting and multifaceted factors were contributing to the low performance. Notably, ‘one-size-fits-all’ approach of the program which was not aligned with the pastorlist community setting population’s livelihood, was the key problem. The findings explicitly indicated that without setting based differentiated approach, community TB program goals, including early case identification can not be achieved. 

### Strength and Limitations of the study

 Data collection by single experienced data collector and efforts made to increase trustworthiness of the analysis and hence of the result, such as members checking, validation of transcripts against audio-recordings and field notes by two independent experts, use of language spoken by the study participants and immediate transcription following the interview were some of the strengths of the study. In addition, participants from all levels of training as community health workers were selected to include all ranges of views. The sex difference between the interviewer and most of the interviewee could have biased the responses. But, the interviewer baracketed himself to minimize the potential bias.

## Conclusions

In this study, we found out that community TB performance is low in the pastoralist community. There are many factors contributing to the low performance and aligning the approach in the context of the pastoralist community setting is suggested as a solution to improve the performance. Therefore, designing and implementing context appropriate approaches such as patient centred and modified DOTs with community treatment supporters is required to achieve the intended goals of the END TB strategy in the pastoral community.

## Data Availability

The data, both audio records and transcripts, analysed during the current study are available from the corresponding author on reasonable request.

## Abbreviations

DOTs: Directly observed short course treatment
HF: Health facility
HP: Health post
TB: Tuberculosis
